# *Salmonella* Typhi–Induced Septic Shock and Acute Respiratory Distress Syndrome in a Previously Healthy Teenage Patient Treated With High-Dose Dexamethasone

**DOI:** 10.1177/2324709616652642

**Published:** 2016-05-30

**Authors:** Melissa Brosset Ugas, Timothy Carroll, Lacey Kovar, Susana Chavez-Bueno

**Affiliations:** 1 University of Oklahoma Health Science Center, Oklahoma City, OK, USA; 2 Acute Disease Service of the Oklahoma State Department of Health, Oklahoma City, OK, USA

**Keywords:** typhoid fever, septic shock, acute respiratory distress syndrome, dexamethasone

## Abstract

Typhoid fever is commonly characterized by fever and abdominal pain. Rare complications include intestinal hemorrhage, bowel perforation, delirium, obtundation, and septic shock. Herein we describe the case of a previously healthy 16-year-old male without history of travel, diagnosed with typhoid fever complicated by septic shock and acute respiratory distress syndrome treated with high-dose dexamethasone. This case details severe complications of typhoid fever that are uncommonly seen in developed countries, and the successful response to high-dose dexamethasone as adjunct therapy. High-dose dexamethasone treatment has reportedly decreased *Salmonella* Typhi mortality, but controlled studies specifically performed in children are lacking, and most reports of its use are over 30 years old and all have originated in developing countries. Providers should include *Salmonella* Typhi in the differential diagnosis of the pediatric patient with fever, severe abdominal pain, and enteritis, and be aware of its potentially severe complications and the limited data on safety and efficacy of adjunctive therapies that can be considered in addition to antibiotics.

## Introduction

*Salmonella enterica* serovar Typhi (*Salmonella* Typhi) causes typhoid fever (TF), a disease that is common in areas with poor sanitation and lack of access to safe food and water.^1^ Approximately 27 million cases of TF are estimated to occur annually worldwide, and in highly endemic areas, infection is more common in infants and preschool children than in older individuals.^2^ *Salmonella* Typhi is transmitted via the oral-fecal route, and after ingestion of the pathogen, a 7- to 14-day asymptomatic period of incubation ensues. Thereafter, fever is the most common symptom. Patients can also have influenza-like symptoms, malaise, and many will experience diarrhea. If untreated, complications such as gastrointestinal bleeding, intestinal perforation, and typhoid encephalopathy can occur, which are closely associated with increased mortality.^3^ Other severe complications such as septic shock or acute respiratory distress syndrome (ARDS) have been recorded but are uncommon. Mortality in many developing countries in the absence of treatment ranges between 12% and 30%.^4^ In the United States, TF is rare, and it is reported mostly among travelers.^4^ Mortality rates are low in the United States, and it is estimated to occur in <0.1% of diagnosed cases.^5^

In this report, we present the case of a 16-year-old male US resident with no history of travel abroad, who developed uncommon life-threatening complications of TF, and responded to a high-dose dexamethasone regimen that is rarely prescribed in the pediatric population.

## Case Report

A previously healthy 16-year-old male was admitted with a 2-day history of abdominal pain, melanotic diarrhea, and fever. On admission, the patient was ill appearing, showed moderate signs of dehydration, and had diffuse abdominal tenderness to palpation. He was tachycardic, but well perfused. The patient had blood and stool cultures performed, he received intravenous (IV) fluids, and was admitted to the general pediatric inpatient service.

Within 12 hours of admission, his blood cultures grew a Gram-negative rod, and the patient was initiated empirically on IV ceftriaxone and ciprofloxacin. On hospital day 1, he developed hypoxia and hypotension that responded initially to fluid resuscitation and supplemental oxygen. However, his respiratory status worsened and he was transferred to the pediatric intensive care unit. The blood culture isolate was confirmed as *Salmonella enterica* serovar Typhi (*Salmonella* Typhi) by the MicroScan WalkAway *plus* System (Siemens Medical Solutions USA, Inc, Malvern, PA). Antibiotic susceptibilities performed with the TREK Sensititre GN4F Gram negative MIC Plate (TREK Diagnostic Systems/Thermo Scientific; Oakwood Village, OH) showed that the isolate was susceptible to ampicillin, trimethoprim/sulfamethoxazole (TMP/SMX), ceftriaxone, azithromycin, and ciprofloxacin. Other laboratory studies revealed a white blood cell count of 3.2 × 10^9^/L, thrombocytopenia with platelets 78 × 10^9^/L, and low fibrinogen of 146 mg/dL. Physical exam showed diffuse lung crackles and a distended, tender abdomen without organomegaly. He did not have peripheral edema or rash.

On hospital day 2, he developed fulminant ARDS with severe hypoxemia and diffuse bilateral infiltrates on chest radiograph. The patient was started on noninvasive positive-pressure ventilation, but despite this intervention his clinical status worsened over the course of 6 hours, and he required endotracheal intubation and mechanical ventilation. He developed hypotension that was not responsive to extensive fluid resuscitation, and he required vasopressor medication for 24 hours. His renal function remained stable throughout his illness.

During his pediatric intensive care unit stay, serial chest radiographs showed worsening pulmonary edema and pulmonary infiltrates bilaterally. Due to his clinical deterioration, TMP/SMX IV was added to his antibiotic regimen with the goal to improve intracellular bacterial killing. Blood cultures became negative within 24 hours of beginning antibiotic therapy, and he became afebrile for the remainder of his hospitalization.

**Figure 1.**
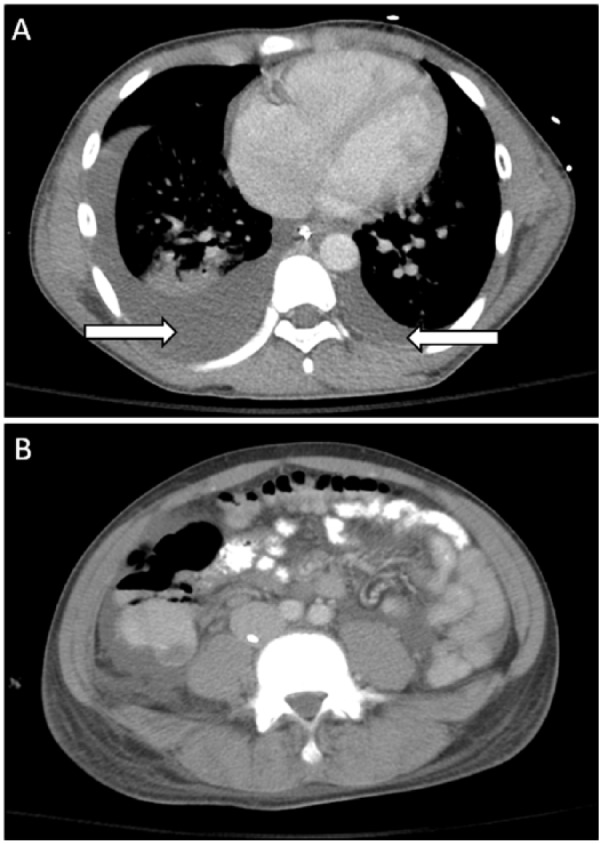
Contrasted computer tomography images of the chest and abdomen demonstrating: (A) large right-sided and small left-sided pleural effusion (arrows), and bilateral confluent/fluffy airspace opacities concerning for ARDS and (B) bowel wall thickening and diffuse ascites without intraperitoneal free air or measurable lymphadenopathy.

On hospital day 4, the patient required increased mechanical ventilatory support with higher inspiratory pressures and greater FiO_2_, and inhaled nitric oxide was started to maintain normal ventilation and oxygenation due to severe ARDS. He developed bilateral transudative pleural effusions that were more severe on the right hemithorax (Figure 1A). An echocardiogram showed normal left ventricular function but elevated pulmonary artery pressures. He required placement of a thoracostomy tube, which was removed after 36 hours. Pleural fluid was sent for culture, and was determined to be sterile. Ciprofloxacin was stopped, and TMP/SMX and ceftriaxone were continued to provide broad coverage for possible nosocomial pathogens in addition to *Salmonella* Typhi infection.

On hospital day 5, the patient developed a rigid abdomen. Contrasted abdominal computed tomography revealed thickened bowel wall with ileal enteritis and severe ascites, but no perforation (Figure 1B). Pediatric surgical consultation was obtained, and no surgical intervention was indicated. He required aggressive diuresis to treat his ARDS, ascites, and pleural effusions. The patient continued to require maximum respiratory support and was not able to be weaned off mechanical ventilation.

Bright red blood was noticed in his nasogastric tube, which was concerning for worsening enteritis since his coagulopathy had already resolved. Due to a lack of overall clinical improvement, he was started on high-dose dexamethasone at an initial dose of 3 mg/kg, followed by 1 mg/kg every 6 hours for a total of 48 hours.^6^ Due to the high-dose steroid therapy, a proton pump inhibitor was added to his regimen to prevent gastrointestinal tract bleeding. The patient clinically improved after completing steroid therapy, and he was successfully extubated on hospital day 10. TMP/SMX was stopped after 6 days of therapy. He completed a 2-week course of ceftriaxone, followed by a week of oral amoxicillin for a total 21 days of antibiotic therapy. He remained in the hospital for a total of 17 days, and he was discharged to his home in good condition.

## Discussion

The clinical features of enteric fever caused by *Salmonella* Typhi vary between geographic regions, but most patients present with influenza-like illness associated with diarrhea and fever, and the majority of patients have a benign course. Humans are the only known reservoir of *Salmonella* Typhi, and transmission is mainly fecal-oral through contaminated foods. Typhoid fever is a reportable disease in the United States, where approximately 400 cases are reported yearly, including 80% in travelers from Southern Asia.^4^ In addition to being a rare disease in this country, severe cases are almost unheard of, and mortality is reported in some studies as zero.^7^

**Figure 2.**
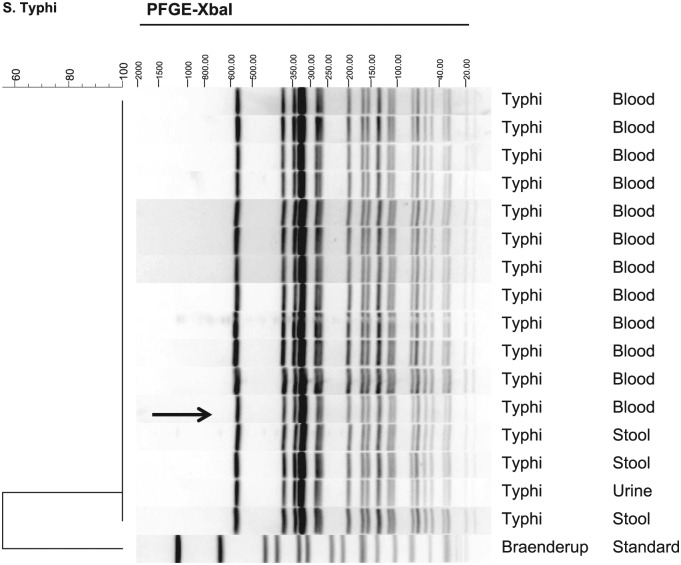
Pulse-field gel electrophoresis (PFGE) image of 16 representative *Salmonella* Typhi isolates from Oklahoman cases including the patient described in this case report (highlighted by the arrow). Digests shown were performed with *Xba*I as the primary restriction endonuclease. PFGE was completed and dendrograms were generated by the Oklahoma State Department of Health Public Health Laboratory.

This case was part of an outbreak of 38 cases of *Salmonella* Typhi investigated by the epidemiologists of the Acute Disease Service of the Oklahoma State Department of Health in Northwestern Oklahoma (NW OK). The outbreak included 23 Oklahomans with culture-confirmed *Salmonella* Typhi, which had an indistinguishable pulsed-field gel electrophoresis (PFGE) pattern (Figure 2). Thirteen outbreak-related cases from NW OK had clinical symptoms consistent with *Salmonella* Typhi, and contact to one or more of the confirmed case(s). Two cases in Iowa residents had *Salmonella* Typhi isolates with indistinguishable PFGE patterns to those from OK cases, and reported travel to NW OK and contact with case families during their incubation period. All Oklahoma cases denied travel away from NW OK during the incubation period, but reported multiple large gatherings that occurred during this time frame. Some of these gatherings were attended by individuals that traveled from outside the United States; our patient attended at least 2 of these community gatherings.

Given the fact that TF is uncommon in the United States, a high index of suspicion is needed for early diagnosis. Blood cultures are the gold standard for diagnosis, although they are positive in only 50% of patients.^4^ Life-threatening complications of TF generally occur after 2 to 3 weeks of illness and may include intestinal hemorrhage or perforation. A fulminant onset of TF manifested by septic shock may occur but is unusual. The main treatment for TF is early antibiotic therapy, which reduces the risk of mortality significantly. Empiric treatment of TF with ceftriaxone or a fluoroquinolone is recommended.^6^ Our patient received broad-spectrum antibiotics initially, but these were then tailored once susceptibilities were available and he demonstrated clinical improvement. Adjunctive therapies such as corticosteroids, in addition to antibiotics, are not standard of care particularly in uncomplicated cases. However, despite broad antibiotic coverage, our patient’s clinical status deteriorated and he developed septic shock and ARDS.

There are no recent reports of ARDS in children with severe TF in the United States, nor on the use of high-dose dexamethasone therapy for life-threatening complications such as this, in pediatric patients in developed countries. Reports of ARDS associated with *Salmonella* Typhi infection have been published in adults, but it is described as a rare TF complication in this population.^8-12^ ARDS is defined by acute onset of respiratory symptoms, a chest radiograph with bilateral infiltrates, and a PaO_2_/FiO_2_ ratio <300 mm Hg (severe hypoxemia), all of which must not be fully explained by cardiac failure or fluid overload.^13,14^ Our patient met all these criteria as his clinical status deteriorated. His hypovolemic shock responded to fluid resuscitation and vasopressors; however, the severity of his ARDS required multiple therapies including high levels of respiratory support to maintain adequate oxygenation.

Our patient’s clinical deterioration prompted the consideration of high-dose dexamethasone as adjunctive therapy. Although corticosteroids have shown improvement in organ function score, lung injury score, and oxygenation in adults with ARDS, treatment with corticosteroids is still controversial, and their use for this indication in children is not routinely recommended.^15,16^ Despite these data, dexamethasone was administered in this case, after careful consideration of the available evidence that support the use of high-dose steroids in TF.

A randomized trial of high-dose dexamethasone (11 mg/kg in 48 hours) or placebo was performed in patients with TF and sepsis.^17^ This study was performed in 1984 in Jakarta, and included children as well as adults (11 out of the 38 patients studied were under the age of 14 years). All patients were treated with chloramphenicol. The case-fatality rate of 10% in the dexamethasone group (n = 20) was significantly lower than the fatality rate of 55.6% in the placebo group (n = 18), with a *P* value of .003. A subsequent study from the same group included 15 additional children with severe TF that received dexamethasone in a nonrandomized fashion. These 2 studies did not show significantly decreased mortality specifically in children who received dexamethasone versus those who did not.^18^ No other recent reports are available that include new data about the use of dexamethasone at such high dose to treat severely ill children with TF. These data are over 30 years old, and this regimen has not been redemonstrated as effective for adults or children in any recent or updated trials in the United States. However, high-dose dexamethasone is still recommended by the American Academy of Pediatrics only in patients that are critically ill due to *Salmonella* Typhi despite being on appropriate antibiotic coverage.^6^ It is postulated that dexamethasone acts as an antioxidant resulting in reduced fatalities, but the exact mechanism of action is not fully understood, nor the safety profile of this regimen.^19^

Our patient received high dose of steroids as specific treatment for complications of severe TF; this is a much higher dose and shorter course than previously studied for ARDS, or for any other acute or severe pathology. Typhoid fever is the only infection where a dose this high is recommended. Given the short duration of therapy, tapering of the dexamethasone regimen was not prescribed. We saw no significant complications with this therapy, and the patient continued to improve clinically soon after steroids were started.

Dexamethasone is unnecessary for most patients with TF but is still recommended for those who are delirious, obtunded, stuporous, comatose, or in shock, for whom this intervention may be life-saving.^6^ This case illustrates the importance of considering TF in the differential diagnosis of patients with fever, abdominal pain, and constitutional symptoms, regardless of their travel history. We also show that despite appropriate antimicrobial therapy, severe complications of TF can ensue. In addition, our case demonstrates a favorable response to high-dose dexamethasone in a pediatric patient with severe septic shock and ARDS due to TF, treated in the United States where many physicians are unfamiliar with the use of this medication at such high dose in children. We recognize that despite this encouraging outcome, the safety and efficacy of this adjunct therapeutic intervention in developed countries is unknown.

In conclusion, TF is rare in the United States, and its potential life-threatening complications are extremely uncommon in children living in developed countries. Despite the limited data on safety and efficacy, high-dose dexamethasone should be considered as adjunctive therapy in severe cases of TF in pediatric patients, after careful consideration of benefits against the risks.
